# Umbilical Cord Blood NOS1 as a Potential Biomarker of Neonatal Encephalopathy

**DOI:** 10.3389/fped.2017.00112

**Published:** 2017-05-22

**Authors:** Jun Lei, Cristina Paules, Elisabeth Nigrini, Jason M. Rosenzweig, Rudhab Bahabry, Azadeh Farzin, Samuel Yang, Frances J. Northington, Daniel Oros, Stephanie McKenney, Michael V. Johnston, Ernest M. Graham, Irina Burd

**Affiliations:** ^1^Integrated Research Center for Fetal Medicine, Department of Gynecology and Obstetrics, Johns Hopkins University School of Medicine, Baltimore, MD, USA; ^2^Aragón Health Research Institute, SAMID Network ref RD12/0026/001, Zaragoza, Spain; ^3^Department of Pediatrics, Johns Hopkins University School of Medicine, Baltimore, MD, USA; ^4^Department of Emergency Medicine, Johns Hopkins University School of Medicine, Baltimore, MD, USA; ^5^Neurosciences Intensive Care Nursery Program, Johns Hopkins University School of Medicine, Baltimore, MD, USA; ^6^Department of Neurology, Johns Hopkins University School of Medicine, Baltimore, MD, USA; ^7^Department of Neurosciences, Kennedy Krieger Institute, Johns Hopkins University School of Medicine, Baltimore, MD, USA

**Keywords:** neonatal encephalopathy, biomarkers, umbilical veins, NOS1, diagnosis

## Abstract

**Background:**

There are no definitive markers to aid in diagnosis of neonatal encephalopathy (NE). The purpose of our study was (1) to identify and evaluate the utility of neuronal nitric oxide synthase (NOS1) in umbilical cord blood as a NE biomarker and (2) to identify the source of NOS1 in umbilical cord blood.

**Methods:**

This was a nested case–control study of neonates >35 weeks of gestation. ELISA for NOS1 in umbilical cord blood was performed. Sources of NOS1 in umbilical cord were investigated by immunohistochemistry, western blot, ELISA, and quantitative PCR. Furthermore, umbilical cords of full-term neonates were subjected to 1% hypoxia *ex vivo*.

**Results:**

NOS1 was present in umbilical cord blood and increased in NE cases compared with controls. NOS1 was expressed in endothelial cells of the umbilical cord vein, but not in artery or blood cells. In *ex vivo* experiments, hypoxia was associated with increased levels of NOS1 in venous endothelial cells of the umbilical cord as well as in *ex vivo* culture medium.

**Conclusion:**

This is the first study to investigate an early marker of NE. NOS1 is elevated with hypoxia, and further studies are needed to investigate it as a valuable tool for early diagnosis of neonatal brain injury.

## Introduction

Neonatal encephalopathy (NE) affects 1–9 out of every 1,000 newborns (1, 2) and has a fatality rate of 9–70% (1, 3, 4); yet, it is still poorly diagnosed at birth. Clinically, NE may manifest as a subnormal level of consciousness or seizures, and is often accompanied by difficulty with initiating and maintaining respiration and depression of tone and reflexes (5). The extent of the brain injury is later assessed by cranial ultrasound and MRI (6). Children diagnosed by clinical and neuroimaging criteria are at an increased risk of developing adverse long-term neurologic sequelae such as seizures and cognitive and motor difficulties (7).

Therapeutic hypothermia is the major medical treatment with promising long-term neurodevelopmental results, and this treatment is effective only when it is administered in the first six postnatal hours (8). Even with this treatment, approximately 40% of patients are affected with sequelae (8). A significant challenge in a timely treatment of NE is that the diagnosis and classification of the severity of the condition are often made only after the brain injury has already occurred. Based on easily accessible material, such as umbilical cord blood, early and prompt diagnosis of NE and associated prognosis would allow early intervention with hypothermia (9), erythropoietin (10), collection of umbilical cord blood for autologous stem cell transplantation (11), or stratification to other emerging therapies. Furthermore, provision of information to the injured neonates’ families, who may face difficult therapeutic decisions, is a key for early intervention.

Given that NE is a rare diagnosis, studies implementing ancillary markers for the diagnosis of the condition are limited. Several biomarkers such as glial fibrillary acidic protein (GFAP), S100 calcium-binding protein B (S100B), neuron-specific enolase (NSE), adrenomedullin, and activin A were investigated, but none of them presented an opportunity for early diagnosis (12–15). A plausible candidate early marker of neurologic injury in neonates is neuronal nitric oxide synthase (NOS1). The association of the enzyme with adverse neurodevelopment is supported by animal studies (16, 17). In addition to neurons, NOS1 expression has been observed in non-neuronal tissues such as skeletal muscle, smooth muscle, and endothelial cells to control muscle contractility and local blood flow (18–20). Furthermore, studies have shown that NOS1 is expressed in cardiomyocytes in response to oxidative stress (21).

We hypothesized that NOS1 in umbilical cord blood is a plausible clinical biomarker for identification of NE due to hypoxic–ischemic events. To test our hypothesis, we pursued four aims: (1) to determine whether NOS1 is present not only in the umbilical cord but also in the umbilical cord blood of neonates; (2) to determine whether the level of NOS1 in neonates with a clinical diagnosis of NE is significantly different from that of neurologically normal neonates; (3) to identify the source of NOS1 in umbilical cord blood; and (4) to simulate hypoxic events that may lead to NE and to evaluate production of NOS1 *ex vivo*.

## Materials and Methods

### Study Design

We undertook a nested case–control study within a prospective cohort of neonates, >35 weeks of gestation; cohort has been described previously (12). Cases were neonates with NE and controls were age-matched newborns, admitted to the Johns Hopkins University Hospital (JHH) Neonatal Intensive Care Unit (NICU) between April 1, 2009 and May 30, 2014 (12). The Institutional Review Board at Johns Hopkins University School of Medicine approved this study, and all participants were anonymized.

Cases (Table 1) were neonates clinically diagnosed with moderate to severe NE, as defined by The American Academy of Pediatrics (5), who underwent whole-body cooling as per the JHH NICU institutional protocol which follows the recommendations of Shankaran et al. (22). Controls (Table 1), matched with the cases by gestational age (within 1 week), were neurologically normal neonates.

**Table 1:** **Maternal demographics and perinatal outcomes for neonatal encephalopathy (NE) cases and normal controls**.

Characteristic	Neurologically normal controls (*n* = 37)	NE cases (*n* = 27)	*P* value
Gestational age at delivery (weeks)	39.6 ± 1.0	39.2 ± 1.4	0.70
Maternal age (years)	26.5 ± 5.7	27 ± 7.6	0.63
Nulliparous	17 (61%)	15 (56%)	0.70
White race	7 (28%)	10 (37%)	0.33
Preeclampsia^a^	3 (11%)	2 (7%)	0.18
Clinical chorioamnionitis^a^	4 (14%)	3 (11%)	0.72
Intrauterine growth restriction^a^	3 (11%)	2 (7%)	0.97
Cesarean delivery	15 (54%)	20 (74%)	0.11
Infant male sex	18 (64%)	17 (63%)	0.92

*Data are presented as means and SD for the two continuous variables, and counts and percentages for the seven categorical variables*.

*^a^Risk factors associated with NE*.

For the secondary analysis, we created a subset that met stringent criteria for NE cases and controls, based on clinical exam plus two additional criteria: umbilical cord arterial pH < 7.0 and base deficit >12 (Table 2) (23, 24). Neurologically normal neonates had a normal exam, umbilical artery pH > 7.2, and base deficit <8.

**Table 2:** **Stringent criteria for neonatal encephalopathy and neurologically normal neonates**.

Encephalopathic neonates	Neurologically normal neonates
At least one clinical criteria Lethargy Stupor or coma Decreased or no activity Distal flexion or complete extension Decerebrate posture Hypotonia or flaccidity Abnormal primitive reflexes Abnormal autonomic nervous system Seizures	Neurologically normal exam
Umbilical cord pH < 7.0	Umbilical cord pH > 7.2
Base deficit >12	Base deficit < 8

### Maternal and Neonatal Characteristics

Maternal and neonatal records were reviewed, and pertinent clinical information was extracted. Data collected included infant sex, mode of delivery, gestational age, maternal age, parity, race, and three conditions suggested being risk factors for NE (25, 26): diagnosis of preeclampsia, clinical chorioamnionitis, and IUGR. Preeclampsia was defined as proteinuria and new onset hypertension. Clinical chorioamnionitis was defined as the presence of maternal fever with at least one other finding of fetal tachycardia, uterine tenderness, or purulent vaginal discharge. IUGR was defined as an estimated fetal weight less than the 10th percentile for gestational age with ultrasound performed within 3 weeks after delivery date.

### Collection of Umbilical Cord Blood for Determination of NOS1

Venous umbilical cord plasma was collected from routinely discarded collected blood. Samples were stored at −80°C until utilized.

### *Ex Vivo* Hypoxia of Umbilical Cord

Umbilical cords were obtained from healthy full-term births within 4 h. Methods previously described by Thomas et al. (27); briefly, in a sterile cell culture hood, umbilical cord was cut into 1-mm length thick slices. For normoxia, specimens were cultured in a conventional incubator preset at 37°C in a humidified atmosphere, containing 5% CO_2_. Hypoxia was induced by a modular incubator chamber. To create gas hypoxia, specimens were first sealed in a humidified hypoxia incubator chamber system (Stemcell Technologies, Vancouver, BC, Canada) supplied with 1% O_2_ at 37°C according to the manufacturer’s instructions. After 3 h of incubation (simulating fetal hypoxic–ischemic event), umbilical cords were fixed with 4% paraformaldehyde (Affymetrix, Cleveland, OH, USA) overnight at 4°C for immunohistochemistry (IHC), or the tissue was trimmed close to the vein and artery followed by immediate storage at −80°C for western blot. Culture media was also collected and frozen at −80°C.

### ELISA for NOS1

ELISA of total NOS1 was performed according to the protocol established in our laboratory for indirect “sandwich” ELISA, optimized for highest sensitivity of human NOS1 in serum, amniotic fluid, or cell lysates. Briefly, an immobilized capture antibody for human NOS1 was coated overnight in 96-well microplate (R&D, Minneapolis, MN, USA). After blocking and sample incubation, the unbound material was washed away, and a biotinylated detection antibody specific for human NOS1 (2 ng/ml, Cell Signaling Tech, Beverley, MA, USA) was used to detect the target protein utilizing a standard streptavidin-HRP format using 3,3′,5,5′-tetramethylbenzidine (TMB) as a substrate and subsequent reading at 450 nm. Biotinylation as well as prior purification of detection antibodies were done using biotinylation and protein A/G purification kits according to manufacturer’s recommendations (Pierce Biotechnology, Rockford, IL, USA). Recombinant purified NOS1 was purchased from Enzo Life Sciences, Inc. (Farmingdale, NY, USA) for standard curve. Average for blank readings was subtracted from the averages for the duplicate readings for each sample, and total NOS1 concentrations of each sample were determined using log-linear regression. ELISA was further validated with JHH population with a separate group of samples.

### Immunohistochemistry

Using a cryostat (Leica, Buffalo Grove, IL, USA), umbilical cords were sectioned into 20-µm thick slices. Sections were incubated overnight at 4°C with primary antibodies in PBS containing 0.5% Triton X-100 (Sigma, St. Louis, MO, USA) after blocking with 10% goat serum. The following primary antibodies were used: rabbit against NOS1 (1:1,000, Millipore, Billerica, MA, USA), NOS2 (1:20, Novus, Littleton, CO, USA), NOS3 (1:100, Abcam, Cambridge, MA, USA), and mouse against CD34 (1:500, Millipore, Billerica, MA, USA). CD34 was used to characterize endothelial cells in human UC. The next day, sections were rinsed with PBS, and then incubated with fluorescent secondary antibodies diluted in 1:500 for 3 h at room temperature. The following antibodies were used for immunofluorescence: goat anti-rabbit DyLight 488 (Abcam, Cambridge, MA, USA) and donkey anti-mouse Alexa Fluor 568 (Life Technologies, Grand Island, NY, USA). The sections were further stained with DAPI (Roche, Indianapolis, IN, USA) for 2 min at room temperature followed by mounting with Fluromount-G (eBioscience, San Diego, CA, USA). Images were attained using Axioplan 2 Imaging system (Carl Zeiss, Thornwood, NY, USA). To exclude a possibility that NOS1 positive cells were blood cells or of stem cell origin, double staining of NOS1/CD44 (Abcam, Cambridge, MA, USA) and NOS1/CD45 (Abcam, Cambridge, MA, USA) was also conducted.

To further confirm the distribution of NOS1 in human UC, another four NOS1 from hosts of rabbit or mouse (Cell Signaling, Beverly, MA, USA; Life Technologies, Frederick, MD, USA; Abcam, Cambridge, MA, USA; and BD Biosciences, Franklin Lakes, New Jersey, USA), one NOS2 (Abcam, Cambridge, MA, USA), and one NOS3 (Abcam, Cambridge, MA, USA) antibodies were utilized to exam the expression together with CD34 antibody. The secondary antibodies were used the same as described above.

### SDS-PAGE and Western Blotting

The following were used as primary antibodies: rabbit against NOS1 (Millipore, Billerica, MA, USA), NOS2 (Novus, Littleton, CO, USA), NOS3 (Abcam, Cambridge, MA, USA) and rabbit anti β-actin (Abcam, Cambridge, MA, USA). Extracted protein (50 µg) was separated by SDS-PAGE using 4–15% gels (Bio-Rad Laboratories, Hercules, CA, USA) and then electro-transferred onto nitrocellulose membranes (Bio-Rad Laboratories, Hercules, CA, USA). Membranes were blocked with 10% BSA (Sigma-Aldrich, St. Louis, MO, USA) in Tris-buffered saline + 0.1% Tween-20 (TBS-T, pH7.5), incubated with primary antibodies in TBS-T containing 10% BSA for 1 h at room temperature, and washed in TBS-T. Goat anti-rabbit IR Dye-800CW (Li-Cor, Lincoln, NB, USA) was used as the secondary antibody. Imaging was performed using the Li-Cor Odyssey Near Infra Red System and analyzed using Image Studio Software (Li-Cor).

### Quantitative PCR (qPCR)

Vein and artery tissue were dissected from umbilical cords and frozen at −80°C until used. The tissue samples were homogenized in RLT buffer (Qiagen, Valencia, CA, USA) for 60 s using a BeadBug microtube homogenizer (Benchmark Scientific, Edison, NJ, USA) with 0.5-mm beads. Cord blood was collected into acid citrate dextrose tubes (BD Biosciences, Franklin Lakes, NJ, USA) and centrifuged at 400 × *g* for 10 min at room temperature. RNA was prepared from the umbilical cord homogenates and cord blood leukocytes with RNEasy Mini Kit (Qiagen, Valencia, CA, USA), and cDNA was prepared using cDNA Synthesis Kit (Bio Rad, Hercules, CA, USA). qPCR was performed in triplicate in 20-µl reactions for 40 cycles, using the manufacturer’s suggested protocols for temperature cycling (Bio Rad, Hercules, CA, USA). The reactions were run on a CFX384 Touch Real-Time PCR Detection System (Bio Rad, Hercules, CA, USA), using SensiFAST Probe No-ROX (Bioline, Taunton, MA, USA). Primers used were obtained from Integrated DNA Technologies (Coralville, IA, USA) for NOS1 (Fwd: AGACGCACGAAGATAGTTGAC, Rev: CCGAAGCTCCAGAACTCAC, Probe:/56-AM/TCCTTAGCC/ZEN/GTCAAAACCTCCAGAG/3IABkFQ), NOS2 (Fwd: ACTTCCACTTGCTGTACTCTG, Rev: CACCTACTTCCTGGACATCAC, Probe: CTGCTGCTCCAAAAGCTGGCC), and NOS3 (Fwd: ACGATGGTGACTTTGGCTA, Rev: TGGAGGATGTGGCTGTCT, Probe: CAGTGGAAATCAACGTGGCCGTG) or SsoAdvanced Universal SYBR Green Supermix (Bio-Rad) for Eukaryotic 18S Ribosomal RNA (Life Technologies, Grand Island, NY, USA). Data analysis was performed with CFX Manager Software (Bio Rad).

### Statistical Analysis

Data were compared using Student’s *t*-test, Mann–Whitney *U* test, or Fisher exact test, when appropriate. Two-tailed Student’s *t*-test or Mann–Whitney *U* test was used subsequently to determine whether data were normally distributed. Fold change of NOS1 level over mean control was calculated.

To determine the ability of NOS1 to predict NE, and the optimal cut points for NOS1, we created receiver operator characteristic (ROC) curves. Sensitivity and specificity were determined for each of these points. Statistical analyses were performed using Stata10 (StataCorp LP, College Station, TX, USA).

## Results

### Study Population

Umbilical cord blood samples of 27 NE cases and 37 controls were used. There were no significant differences between cases and controls in: (i) maternal demographic parameters (gestational age at delivery, maternal age, nulliparous, and white race), (ii) incidence of adverse perinatal outcomes (preeclampsia, clinical chorioamnionitis, intrauterine growth restriction), or (iii) cesarean delivery or infant sex (Table 1). For an exploratory binding assay to detect NOS1 in umbilical cord blood of neonates, 15 NE and 24 control neonates were utilized. Eight neonates met stringent criteria for NE and were matched to ten control neonates (Table 2). Another 12 NE and 13 controls were utilized to validate the exploratory results by ELISA. We demonstrated the presence of NOS1 in the umbilical cord blood of all 64 neonates.

### Level of NOS1 in Neonates with NE and in Neurologically Normal Neonates

**Figure 1:**
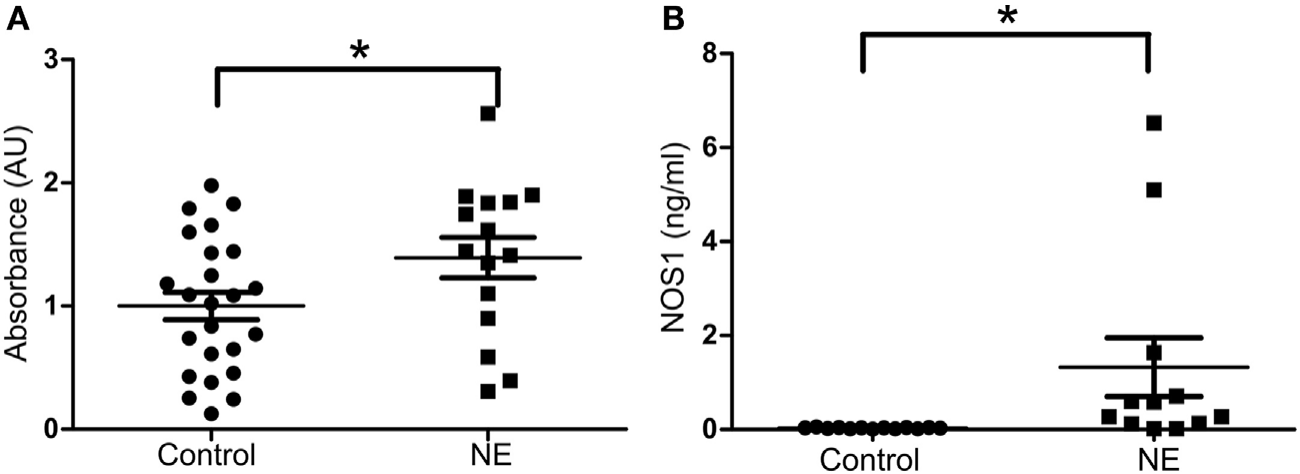
**Expression of NOS1 in umbilical cord blood**. **(A)** Bar plot of relative absorbance in NOS1 level compared to controls based on exploratory NOS1-binding assay (*n* = 15 NE cases and 24 controls, Student’s *t*-test; *p < 0.05). **(B)** NOS1 concentrations were determined by ELISA using a separate set of patients (*n* = 12 NE cases and 13 controls; Student’s *t*-test; *p < 0.05). Data are reported as means ± SEM.

In our investigational study, as determined by relative absorbance values of ELISA (Figure 1A), the level of NOS1 in umbilical cord blood was significantly higher in NE cases (mean = 1.392 AU, 95% CI 1.04–1.74) than in the controls (mean = 1.00 AU, 95% CI 0.77–1.23). Since these values were generated from a non-commercial ELISA (prepared in our laboratory and originally without standards/standard curve), these findings were validated by a separate experiment to determine NOS1 concentration, involving separate set of samples and standards/standard curve (NE: mean = 1.32 ng/ml; Control: mean = 0.03 ng/ml; *p* < 0.05, Student’s *t*-test; Figure 1B).

### Stringent Criteria Subset

**Figure 2:**
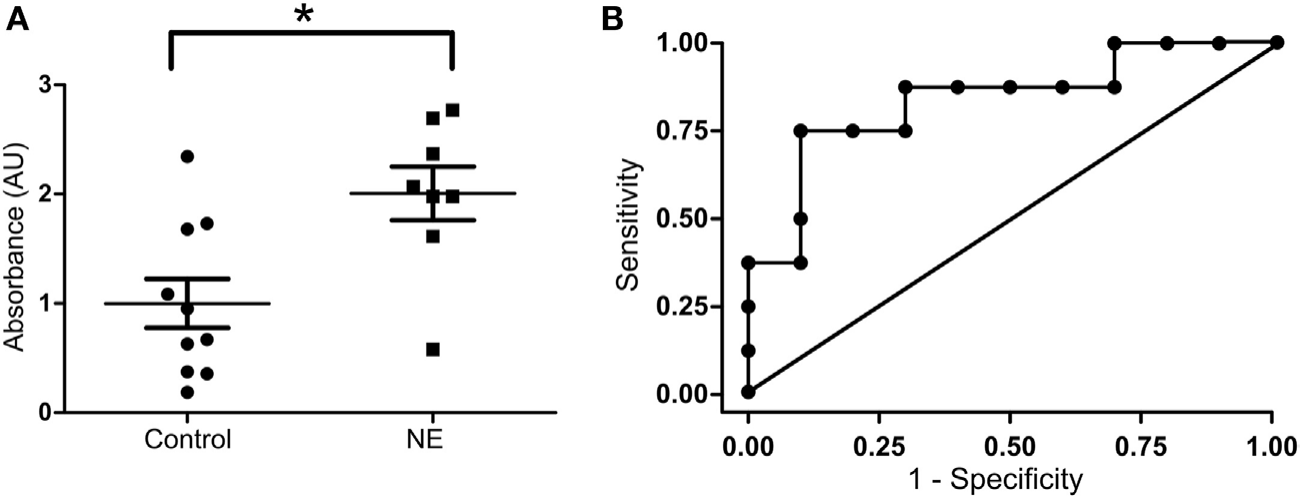
**NOS1 expression in NE patients classified by stringent criteria**. **(A)** Relative absorbance of NOS1 expression compared to controls based on exploratory NOS1 binding assay (*n* = 8 NE cases and 10 controls, Student’s *t*-test; *p < 0.05). Bars represent mean ± SEM. **(B)** Receiver operator characteristic (ROC) curve for NOS1 expression. Area under the curve equals 0.84.

In umbilical cord blood, the mean relative absorbance of NOS1 level was significantly higher (Figure 2A, *p* < 0.05, Student’s *t*-test) in the neurologically at-risk neonates meeting stringent criteria (mean = 2.006 AU, 95% CI 1.425–2.587) than in the neurologically normal control neonates (mean = 1.000 AU, 95% CI 0.493–1.507). Within the group of neonates meeting stringent criteria, NOS1 was predictive of NE. The ROC curve yielded an AUC of 0.84 for NOS1 prediction of NE (Figure 2B).

### NOS1 Expression in Endothelial Cells of Human Umbilical Vein

**Figure 3:**
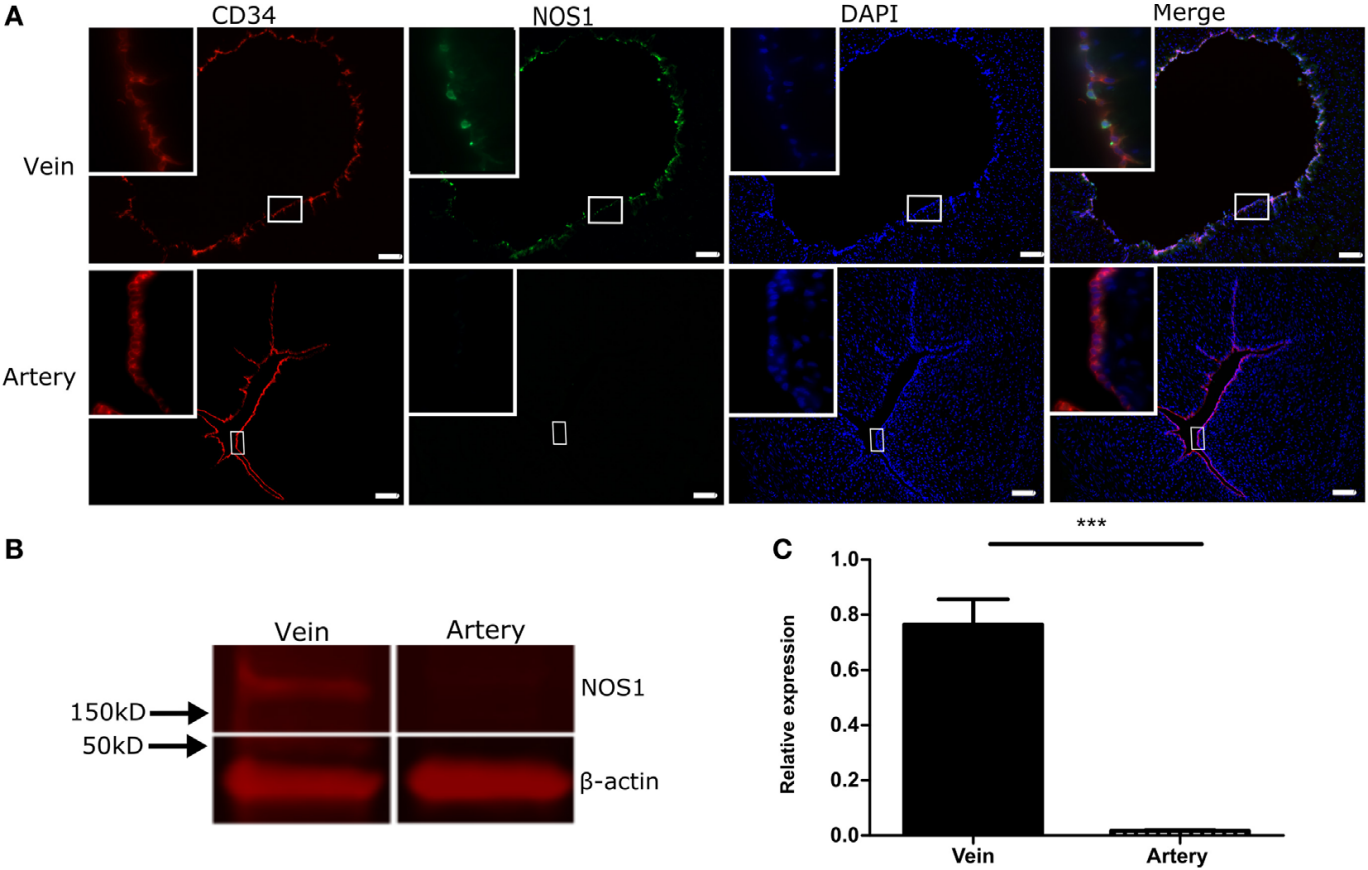
**NOS1 Expression in vessels of human umbilical cord**. **(A)** Immunostaining of CD34 and NOS1 in endothelial cells of vein and artery of umbilical cord (*n* = 4 per group). **(B)** Protein expression of NOS1 in vein and artery of umbilical cord by western blot. **(C)** Densitometry analysis of western blot imaging (*n* = 5 per group; Student’s *t*-test; *p < 0.05). Scale bars = 100 µm.

To localize NOS1 in the human umbilical cord, IHC for NOS1 and CD34 were performed. IHC for CD34 revealed a distribution of endothelial cells in the umbilical cord (28). NOS1 was expressed mainly in the form of fine granules in the cytoplasm and, occasionally, in cell membrane. There was a co-localization of NOS1 and CD34 in the umbilical vein, as demonstrated by double-positive staining (Figure 3A, top panel). Neither CD44 nor CD45 cells was co-localized with NOS1 (data not shown). There was no clear NOS1 expression in umbilical artery (Figure 3A, bottom panel). Western blot further confirmed IHC results with significant expression of NOS1 in umbilical vein (Figures 3B,C, Student’s *t*-test, *p* < 0.05).

Confirmatory analysis examining expression of NOS1, NOS2 (inducible NOS), and NOS3 (endothelial NOS) at mRNA and protein level in umbilical cord and leukocytes were also performed. NOS1 was only detected in umbilical cord vein (Table 3), while NOS2 and NOS3 expression was detected by IHC and qPCR in both umbilical cord vein and artery. Transcripts for NOS1, NOS2, and NOS3 were not detected in cord blood leukocytes (Table 3) using qPCR.

**Table 3:** **Detection of NOS1, NOS2, and NOS3 in umbilical cord vein and artery**.

	Immunohistochemistry (IHC)			Quantitative PCR		
	NOS1	NOS2	NOS3	NOS1	NOS2	NOS3
Umbilical cord artery	− (0/4)	+ (4/4)	+ (4/4)	− (0/4)	+ (4/4)	+ (3/4)
Umbilical cord vein	+ (4/4)	+ (4/4)	+ (4/4)	+ (3/4)	+ (4/4)	+ (3/4)
Cord blood leukocytes				− (0/4)	− (0/4)	− (0/4)

*−, negative expression; +, positive expression. There were four samples for each group. In brackets, numerators are the numbers tested negative or positive expression by IHC or quantitative PCR*.

To further ascertain a differential expression as well as to validate the specificity of the antibodies used, another four NOS1, one NOS2, and one NOS3 antibodies were used in IHC. Similar to the above results, NOS1 expression was detected in umbilical cord vein but not in artery (Figure S1A in Supplementary Material). NOS2 (Figure S1B in Supplementary Material) and NOS3 (Figure S1C in Supplementary Material) were detected in both umbilical cord vein and artery.

### Western Blot and ELISA Analyses of NOS1 Expression in *Ex Vivo* Umbilical Cord Culture

**Figure 4:**
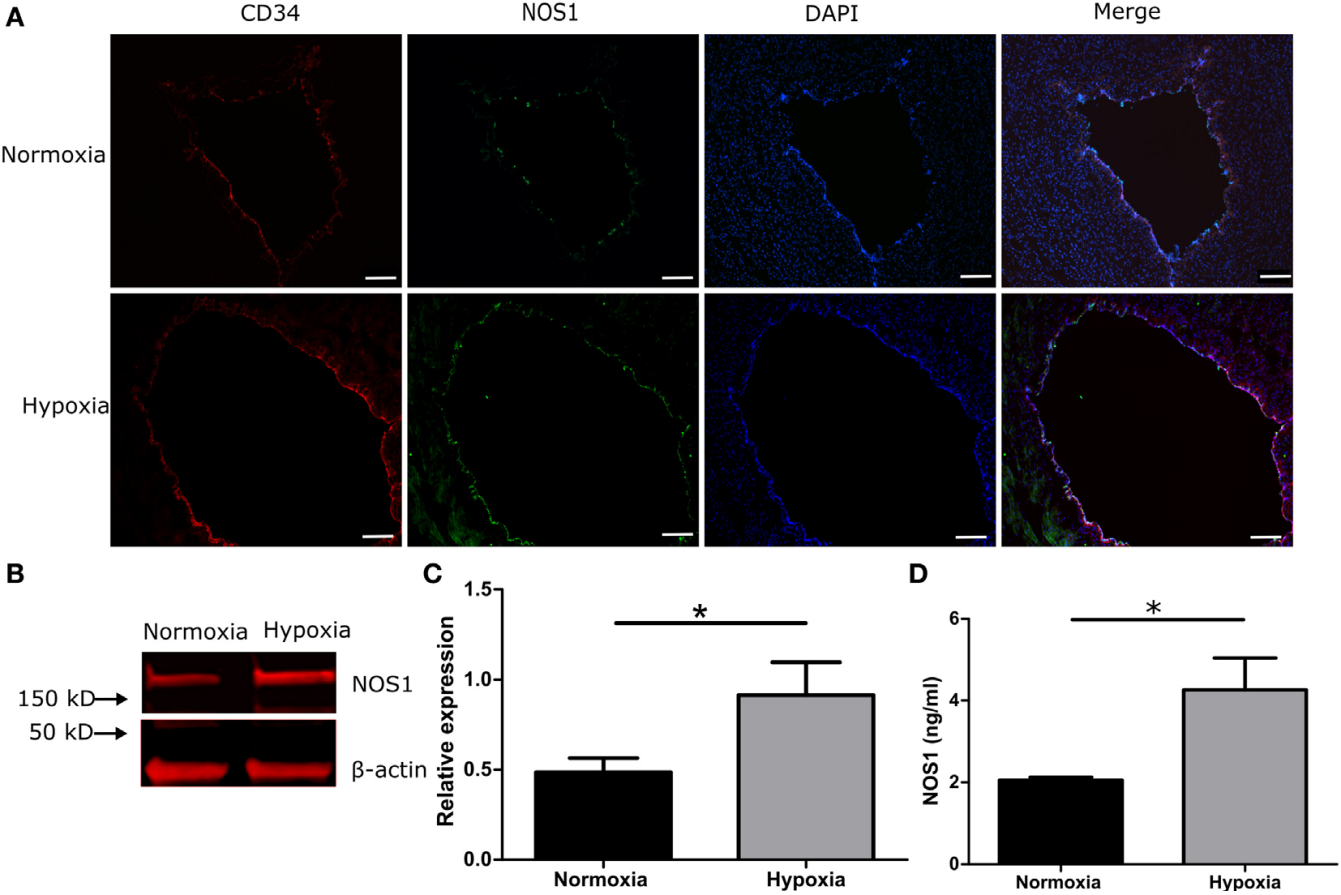
**NOS1 expression in endothelial cells of human umbilical vein in 1% hypoxic chamber**. **(A)** Immunostaining of CD34 and NOS1 in vein (*n* = 5 per group). **(B,C)** Protein levels of NOS1 in umbilical cord by western blot (*n* = 5 per group; Student’s *t*-test; *p < 0.05). **(D)** NOS1 production in *ex vivo* culture supernatant by ELISA (*n* = 5 per group; Student’s *t*-test; **p < 0.01). Scale bars = 10 µm.

To investigate whether hypoxia induces a NOS1 response in venous endothelial cells, umbilical cord slices were cultured in a hypoxia chamber under 1% O_2_. After 3 h of culture (simulating a clinical hypoxic event), the co-localization of CD34 and NOS1 increased in the hypoxic group compared with normoxic control, by IHC (Figure 4A) and by western blot (Figures 4B,C, *p* < 0.05, Student’s *t*-test). We further measured NOS1 production in culture supernatant and, after 3 h of hypoxia, NOS1 increased significantly following hypoxic conditions (Figure 4D, Student’s *t*-test, *p* < 0.01).

## Discussion

Our study is the first to demonstrate that NOS1, a marker of oxidative stress, is a potential early bedside clinical biomarker of NE in umbilical cord blood. In our study, the levels of NOS1 were significantly higher in the umbilical cord blood of neonates with clinical encephalopathy than in normal controls. Furthermore, our *ex vivo* experiments confirmed that NOS1 was increased with hypoxia in venous endothelial cells of umbilical cord.

There are a few biomarkers relative to neonatal brain injury. NOS1 is currently the only early biomarker that is available to aid in diagnosis of NE at delivery based on umbilical cord blood. Massaro et al. evaluated the serum biomarkers S100B and NSE in neonates treated with hypothermia and found them to be associated with clinical encephalopathy and MRI change (3), consistent with prior studies (13–15, 29). None of the biomarkers were used for diagnosis or triage of neonates to therapy. In addition, the first time point evaluated was at initiation of cooling; information was not available prior to initiation of cooling and could not assist with triage to therapy which represents a limitation of their study. Furthermore, others evaluated adrenomedullin and activin A have been evaluated as biomarkers of NE in neonatal serum but similarly have not been tested in umbilical cord blood (30). In Ennen’s studies (12), GFAP in serum at 24 and 48 h after birth was predictive of brain injury on MRI in encephalopathic neonates. Similarly to other markers discussed, at birth, umbilical cord blood GFAP levels were not predictive of brain injury.

We used ELISA for NOS1, which may present as a new, fast, and easy method to perform clinical test due to high levels (nanograms) of NOS1 presented in umbilical cord blood. As the AUC of NOS1 test is perhaps superior as compared to current clinical criteria for diagnosis of NE [AUC 0.81 (31)], we speculate that implementation of this test will improve sensitivity and specificity of timely clinical diagnosis as it can be used concomitantly. Results of this test can be provided within the first several hours of life, allowing for early diagnosis and triage to therapy such as hypothermia and other novel therapeutics. One novel use for such clinical test would include early identification of which neonates that might benefit from storage of umbilical cord blood for later stem cell transplantation and would result in timely collection of umbilical cord blood.

Neonatal encephalopathy is a rare diagnosis and MRI diagnosis of actual brain injury in this group is an infrequent finding. The consequences of NE including death and neurodevelopmental disability can be devastating for the child and family. While one critique of our study could be a small sample size, the nested case–control study represents a valuable sample, given that NE is rare, even in our referral university center. However, our data are confirmed with simulation of hypoxia *ex vivo*, and this is an advantage of our study. Further research will concentrate on collecting information about childhood outcomes of these at-risk neonates with neurodevelopmental testing at 1–3 years of age.

Our study has several strengths. An ELISA assay quantified the level of NOS1, and this test can be used in a clinical setting to inform clinical decisions early in neonatal life. The results are reproducible, and more stringent criteria for encephalopathy yields improved prediction of NOS1 for NE. In addition to identifying neonates at risk of brain injury, a biomarker would timely predict which infants are at risk for brain injury and which are most likely to have long-term neurologic sequelae. Moreover, our study identifies a source of NOS1, and, using experimental conditions that mimic short-term hypoxic environments, we are able to observe similar findings *ex vivo*.

Perinatal hypoxia–ischemia is an important cause of NE (50–80%) (32). Nitric oxide signaling has been demonstrated to have an important role in hypoxia-induced pathological responses as all NOS isoforms critically require oxygen for activity (33). Physiologically, NOS1 depends more on oxygen and has higher rates of nitric oxide biosynthesis (34) than NOS2 and NOS3. Both NOS1 mRNA and protein expression are regulated by O_2_ at transcriptional and translational level (35, 36), and therefore, it may be more sensitive to hypoxia. Corollary to that, in our study, NOS1 significantly increased in umbilical cord blood of moderate and severe NE neonates compared to control.

To investigate a plausible origin of NOS1 in umbilical cord blood, we performed a survey of all possible sources of NOS1 in umbilical cord blood and umbilical cord. We found that NOS1 was present in endothelial cells of human umbilical cord vein but not in umbilical cord artery. While our results are in keeping with a previous study (20) that showed NOS1 expression in vein of human umbilical cord by IHC, our study is the first to demonstrate a differential expression of this enzyme in umbilical vein and artery. In the adult vascular system, NOS1- and NOS3-derived nitric oxide is thought to play a major role in changes of vascular arterial tone (37, 38). In addition to regulating vascular tone, nitric oxide is an important signaling molecule in the nervous system and the immune system (39, 40). We speculate that endothelial NOS1 expression in umbilical vein may be a conserved mechanism that contributes to the fetal response to hypoxia and inflammation. Further studies to clarify the role of umbilical cord NOS1 during hypoxic and inflammatory conditions are needed.

In conclusion, we speculate that NOS1 is a novel, early biomarker for early identification of NE that can be used in determining NE together with clinical manifestations to improve diagnosis, allowing for better prognostic information and earlier interventions.

## Ethics Statement

This study was carried out in accordance with the recommendations of Johns Hopkins University Hospital (JHH) Neonatal Intensive Care Unit and the protocol was approved by The Institutional Review Board at Johns Hopkins University School of Medicine. All participants were anonymized.

## Author Contributions

JL and JR performed the experiments, analyzed data, and wrote the manuscript. SY analyzed data and wrote the manuscript. RB and SM perfumed the experiments and analyzed data. CP, EN, AZ, FN, DO, EG, and MJ designed the experiments and wrote the manuscript. IB designed and performed the experiments, analyzed data, and wrote the manuscript.

## Conflict of Interest Statement

The authors declare that the research was conducted in the absence of any commercial or financial relationships that could be construed as a potential conflict of interest.

## Supplementary Material

Figure S1 **Distribution of NOS1, NOS2, and NOS3 expression in umbilical cord**. **(A)** Representative image of NOS1 (green) in umbilical cord using four different antibodies. NOS1 was expressed in the venous endothelial cells (red) of human umbilical cord, but not in the artery. **(B)** NOS2 was expressed in both endothelial cells (red) of vein and artery. **(C)** NOS3 (green) was also expressed in both endothelial cells (red) of vein and artery. Scale bars = 10 µm.
