# Supplementary figures and images for: Umbilical Cord Blood NOS1 as a Potential Biomarker of Neonatal Encephalopathy
Source: Front Pediatr. 2017 May 22;5:112. doi: 10.3389/fped.2017.00112 (PMC5466059; doi:10.3389/fped.2017.00112)

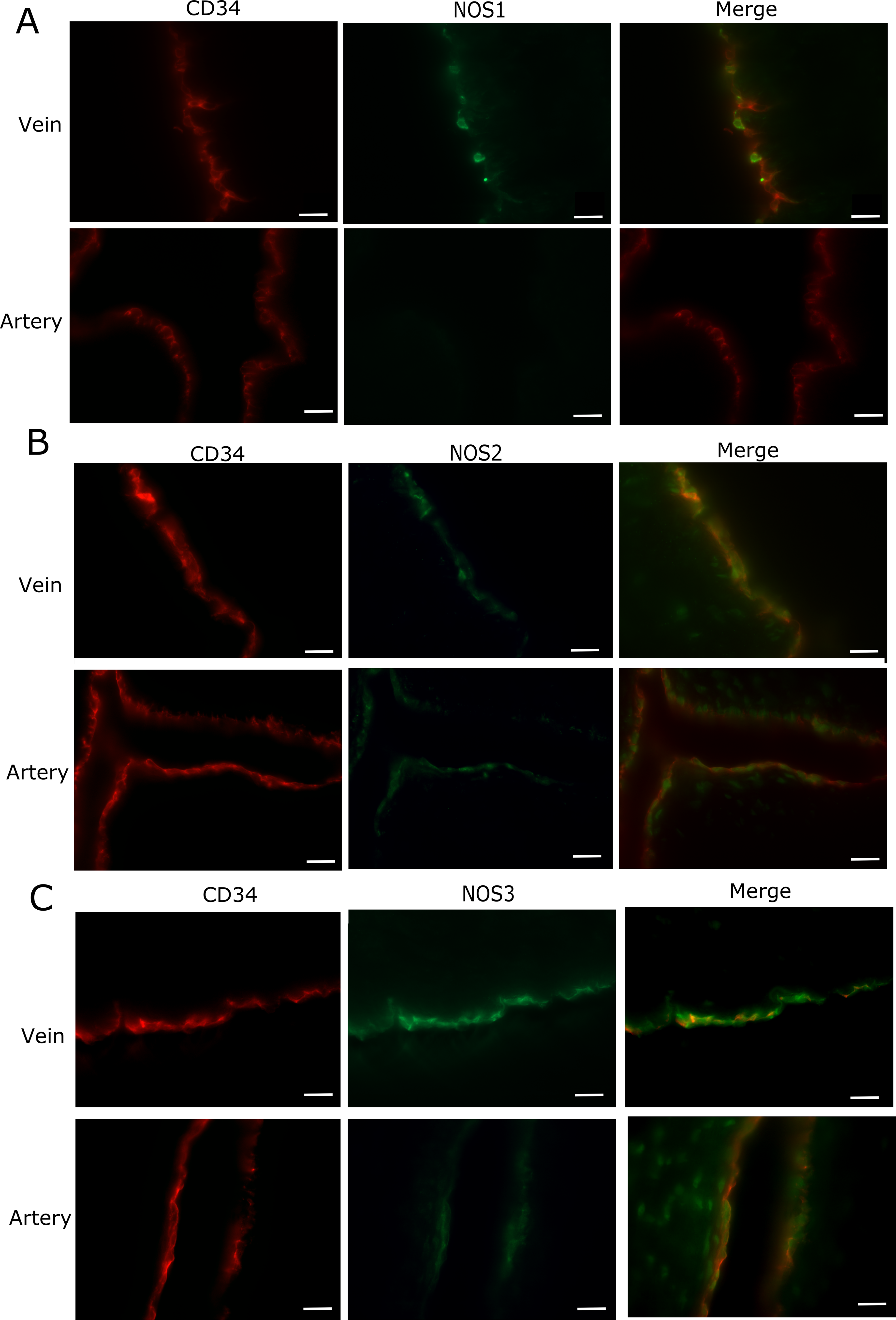

Supplement: Figure S1 — Distribution of NOS1, NOS2, and NOS3 expression in umbilical cord. (A) Representative image of NOS1 (green) in umbilical cord using four different antibodies. NOS1 was expressed in the venous endothelial cells (red) of human umbilical cord, but not in the artery. (B) NOS2 was expressed in both endothelial cells (red) of vein and artery. (C) NOS3 (green) was also expressed in both endothelial cells (red) of vein and artery. Scale bars = 10 µm. [file Image_1.TIFF]
